# A Case of Hypernatremia With Dementia

**DOI:** 10.7759/cureus.39603

**Published:** 2023-05-28

**Authors:** Drashti Desai, Shefali Pati, Ma. Carla Angela Evangelista

**Affiliations:** 1 Internal Medicine, New York City Health and Hospitals Corporation (NYC HHC) Lincoln Medical Center, New York City, USA

**Keywords:** geriatrics, nephrology, medicine compliance, palliative, desmopressin test, hypernatremia, chronic lithium therapy, bipolar disorder (bpd), progressive dementia, nephrogenic diabetes insipidus

## Abstract

The authors report a case of hypernatremia in a patient with a history of dementia. This case highlights the challenges and scope of taking care of such patients. It also highlights the hardships in diagnosing and caring for patients with inadequate documentation of past diagnoses and treatments.

## Introduction

Hypernatremia occurs when the serum value of sodium is greater than 145 mEq/l. Hypernatremia can cause brain shrinkage, resulting in vascular rupture and intracranial bleeding [1]. It is invariably marked as hypertonic hyperosmolality and always causes cellular dehydration. Primary water deficit is one of the significant causes of hypernatremia, often caused due to inadequate access to water or dysfunction of the thirst mechanism. Medications can also cause hypernatremia, commonly including phenytoin, lithium, and amphotericin, among others, profoundly affecting the secretion of the anti-diuretic hormone and, thus, the effect on water balance and osmolality.

Cognitive dysfunctions seen in dementia impair a person’s ability to perform self-care tasks, reduce their ability to communicate, and make the goals of care more challenging for the patient and the caregiver. In this case report, the authors report a 56-year-old woman with dementia who could not communicate, understand, or follow commands. She was brought in from her nursing home with hypernatremia and hypoactive delirium. Considering the patient was found drinking urine from a patient's Foley bag, her thirst center was intact, but she could not communicate. This case report delves deeper into healthcare workers' challenges while managing such patients.

## Case presentation

The patient was a 56-year-old female with a history of hypertension, coronary artery disease, chronic kidney disease, chronic hypernatremia since 2018, bipolar disorder with psychotic features, catatonia, bilateral hand tremors admitted for increased aggressiveness and agitation while being found drinking urine from another patient's urine bag and wandering with an unstable gait. The patient had past admissions for catatonia and neuroleptic malignant syndrome and was on quetiapine 50 mg orally twice a day, risperidone 1 mg orally nightly, olanzapine 5 mg nightly, lurasidone 40 mg daily, and amlodipine 5 mg tab daily in the nursing home since 2020. The history of compliance with medications before 2020 was unknown. She has no surgical history and no allergies to medications. She also had a history of smoking tobacco for approximately 25 years with unknown packs and alcohol use for 25 years intermittently, and cocaine for 15 years but not daily. She also had a family history significant for alcohol use and bipolar disorder in the father. The patient was baseline aggressive in nature, but it had worsened in severity in the last three to four weeks.

**Figure 1:**
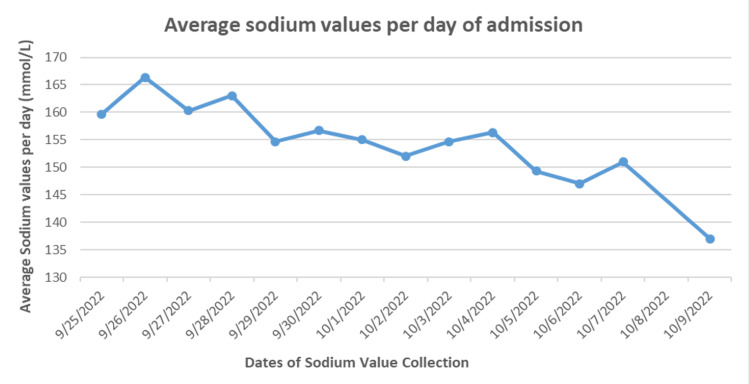
Trends of sodium per day of admission

The patient had difficulty making her needs known and was in a wheelchair due to impaired mobility. Psychiatry was consulted, and they diagnosed her with delirium. On admission, the patient was oriented to name only and partially followed commands. She was confused and could not speak full sentences or words. While inpatient, she was started on amlodipine 5 mg oral, enoxaparin 30 mg subcutaneous, insulin lispro 0-6 units, melatonin 1 mg, quetiapine 50 mg nightly, risperidone 1 mg nightly, and cogentin 1 mg tablet. On exam, significant vitals included a BMI of 17 kg/m^2^ and BP of 145/82 mmHg. When offered, the patient drank almost four to five cups of water voluntarily. The patient showed dry mucous membranes, decreased skin turgor, dry lips, and skin with weight loss. Labs showed hypernatremia (158 mmol/L), blood urea nitrogen (BUN; 49 mg/dL), and creatinine( 2.13 mg/dL), which was above her usual baseline ( Na 150 mmol/L). She had significant polyuria with 24-hour urine output showing 1750 ml urine. She also had an increased serum osmolarity ( 340 mOsm/kg) with low-normal urine osmolarity (150 mOsm/kg) and urine specific gravity of 1.006. Lithium levels were negative. Ultrasound of the kidneys showed small kidneys with increased echogenicity bilaterally. The water deficit was calculated as 2.82 liters with Na 162 mmol/L. The patient was started on half liter IV bolus of normal saline and was placed on dextrose 5% water for maintenance at 100 ml/hr and was found to have persistent hypernatremia despite being given fluids. On the next day, her sodium kept trending upward (162 mmol/L), and endocrine was consulted. At this time, no input/output data was available. A water deprivation test was done and desmopressin was used to establish a diagnosis of nephrogenic diabetes insipidus (DI) (Table 1). The patient was then started on hydrochlorothiazide 25 mg, supplemented with an increase in oral fluid intake of at least one-two glasses of 250 ml of water every two hours, and sodium decreased from 160-152 in 10 hours. The patient was continued on these medications, aiming to keep sodium correction at the rate of 8-10 mEq/L in 24 hours (Figure 1). This allowed the IV fluids to be discontinued. The patient could not verbally ask for water, nor could she communicate about her needs due to dementia and overall cognitive decline. So caregivers on floors were instructed to watch out for nonverbal cues of thirst. Sodium had been observed to stabilize with noticeable improvement of mental status back to a documented baseline from the facility. The patient's hospital stay was for approximately three months and was discharged on home medications with the addition of hydrochlorothiazide 25 mg with close monitoring of vitals and patient mental status. Facilities were instructed about the patient’s special needs. The patient was eventually discharged back to a nursing home.

**Table 1:** Urine osmolarity at baseline versus at every 30, 60, and 90 minutes

	Baseline (mmol/L)	30 mins (mmol/L)	60 mins (mmol/L)	90 mins (mmol/L)
Urine Osmolarity	172	176	183	184

## Discussion

One of the main problems in the aforementioned case was hypernatremia in patients with dementia. Hypernatremia occurs when the serum value of sodium is greater than 145 meq/L.

In this case, many etiologies together could have contributed to the patient’s hypernatremia such as poor water intake due to dementia, underlying psychiatric symptoms, and chronic use of lithium. When the patient was brought in with her condition, medical records showed documentation that the patient had been on lithium in the past, however, a formal diagnosis of DI was not found on her chart. So the main focus was to see if we could formally diagnose her with DI and categorize it, thus warranting a water deprivation test and administering desmopressin to assess the responsiveness of the V2 receptor.

As seen in Table 1, the urine osmolality measured after administration of the desmopressin test failed to show a rise of > 45% or a value >300 mOsmol/kg, consistent with the criteria for complete nephrogenic DI. Nephrogenic DI develops when the collecting duct does not respond to the anti-diuretic hormone. Aquaporin-2 water channels do not appropriately insert into the collecting duct to reabsorb water, resulting in constitutively diluting urine due to chronic free water loss.

Lithium can cause nephrogenic DI in up to 20-40% of patients taking the medication [2], and a subset of these patients will have a persistent concentrating defect long after lithium is discontinued. This condition can occur up to eight years after discontinuing lithium. Patients with nephrogenic DI are at risk for serious hypernatremia when fluid intake is restricted for any reason. The mechanism of acute lithium-induced nephrogenic DI, while the patient is on lithium, is related to changes in intracellular cyclic adenosine monophosphate; there are some reports in which lithium damages the mRNA responsible for aquaporin-2 channel production [3,4].

The usual management of patients with nephrogenic DI includes dietary changes and increased consumption of water; low-dose thiazides lead to a mild reduction in plasma volume, which activates the renin-angiotensin-aldosterone system (RAAS). The increased AT2 production increases proximal tubular reabsorption of sodium and water; this is why thiazides reduce the obligate amount of filtration, therefore reducing urine output [4,5]. This patient was given hydrochlorothiazide and prompted water intake after initial IV fluid correction with D5W (dextrose 5% in water), achieving the patient's previous baseline sodium level. The importance of monitoring sodium correction is essential since it comes with the consequences of developing neurological complications if sodium is overcorrected too rapidly [6-9].

Dementia is a serious decline of global cognitive ability in a previously unimpaired person beyond what might be expected from normal aging. Environmental factors, including a change of routine, overstimulation, isolation, inadequate temperature, and not being able to make needs known, can trigger the behavioral and psychological symptoms of dementia. This patient's dementia made it harder for her to communicate. The affected cognitive areas were loss of memory, attention, language, and problem-solving. This communication gap made it hard for healthcare professionals to get her immediate needs met. But having an intact thirst center allowed her to show non-verbal thirst cues such as getting any source of liquid that she could get her hands on and drinking water readily when given. Physicians can perhaps anticipate and identify a baseline volume of daily water intake as a goal of care for complicated patients such as this one. Further, they can monitor daily weights and/or quantify urine output. Other professionals, such as nurses and dieticians, can work holistically to reach the goals of such patients [10].

Dementia-associated cognitive impairment affects a person's capacity to take charge of their own care, including taking responsibility for their medications and checking their blood sugar [11]. It is also linked to higher usage of healthcare and social services [12]. Additionally, older adults with dementia are more likely to experience hypoglycemia than persons without dementia [13], and polypharmacy may increase the risk of drug interactions and serious adverse reactions [14]. Additionally, there is research to suggest that those with dementia may have less access to diabetic services and monitoring than individuals without dementia [14].

It's critical to develop strategies for managing diabetes in elderly individuals and those with complicated medical requirements, including issues like tailoring glycemic targets for individuals with dementia and developing strategies that consider this population's vulnerability to and the effects of hypoglycemia [15]. People who have diabetes usually use patient-centered methods to establish glycemic control that weigh treatment goals against quality-of-life considerations along with patient and caregiver preferences [16] and include patients and caregivers in the formulation of treatments. Theories concerning how services are developed or put into practice for those with dementia should be encouraged, such as the work done by clinical staff in customizing or collaborating on interventions for individuals, including implementing case management strategies [15,16].

Dementia may become clinically dominant and interfere with the management of illnesses like DI if it is linked to difficult behaviors (such as hostility, agitation, or psychosis) [17]. Interventions should be placed that help family caregivers of dementia patients deal with the behavioral, psychological, and emotional effects of dementia [18] through education and support. Identification, management, and prevention of long-term consequences, such as depression, visual issues, and neuropathic difficulties, in patients with dementia should be the focus of future research. There should be an emphasis on the reduction of potential side effects in patients with dementia with poorly controlled diabetes such as falls, blindness, vascular problems, and kidney failure. Compliance with medications in these patients, diabetes management, assistance for older populations, multi-morbid seniors with dementia, and treatments involving family caregivers of patients with dementia are a few examples of interventions.

The execution and acceptance of interventions intended to improve the physical well-being of people with dementia should be studied for both obstacles and advantages (e.g., dementia-friendly campaigns, the impact of the cognitive versus behavioral and psychological symptoms of dementia, and the course of dementia on family caregivers and service providers). Research presenting opportunities for convertible learning, including those that analyze how services are provided and carried out for people with dementia (for instance, interventions to improve access or continuity of care) should be encouraged.

## Conclusions

Medicine compliance is an important benchmark to achieve in patients with dementia, which proves harder without a multidisciplinary team’s involvement and timely dosages. This case report engages and opens up possibilities for further studies in care for patients with dementia with nephrogenic diabetes insipidus whose management with thoughtful medical care is crucial. The measurement of daily weight, blood pressure, pulse, urine output, and polyuria are important markers. Furthermore, physical examinations, such as skin turgor and mucus membrane moisture, are also critical in a patient who is unable to communicate fluid/nutritional needs. But treatment for medically complicated patients with dementia does not just center on pharmacological aspects alone but also on the awareness of the caregiver in recognizing non-verbal communications from such patients to provide holistic care for such patients.
